# Cooperativity of α-Synuclein Binding to Lipid Membranes

**DOI:** 10.1021/acschemneuro.1c00006

**Published:** 2021-06-02

**Authors:** Katarzyna Makasewicz, Stefan Wennmalm, Björn Stenqvist, Marco Fornasier, Alexandra Andersson, Peter Jönsson, Sara Linse, Emma Sparr

**Affiliations:** † Division of Physical Chemistry, Department of Chemistry, Lund University, P.O. Box 124, SE-22100 Lund, Sweden; ‡ Department of Applied Physics, Biophysics Group, SciLifeLab, Royal Institute of Technology-KTH, 171 65 Solna, Sweden; § Division of Biochemistry and Structural Biology, Department of Chemistry, Lund University, SE-22100 Lund, Sweden

**Keywords:** Cooperative binding, homotropic allostery, α-synuclein, lipid membrane, Adair equation, fluorescence correlation spectroscopy

## Abstract

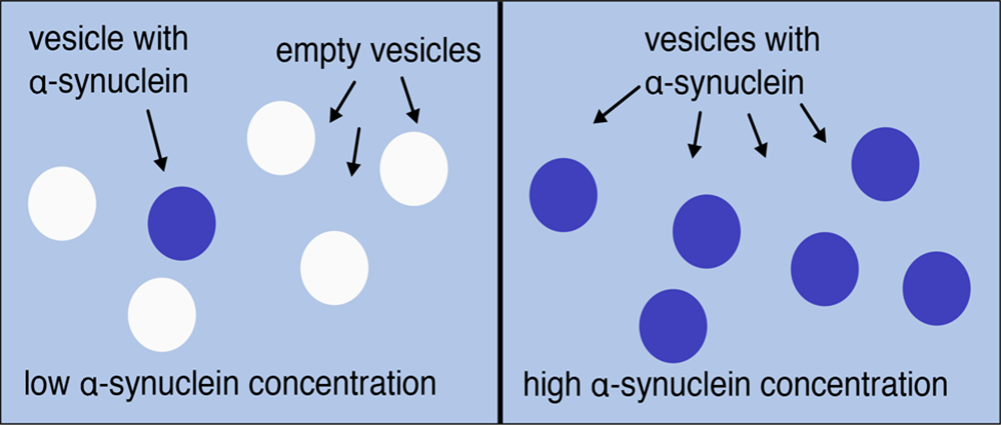

Cooperative binding is a key feature of metabolic pathways, signaling, and transport processes. It provides tight regulation over a narrow concentration interval of a ligand, thus enabling switching to be triggered by small concentration variations. The data presented in this work reveal strong positive cooperativity of α-synuclein binding to phospholipid membranes. Fluorescence cross-correlation spectroscopy, confocal microscopy, and *cryo*-TEM results show that in excess of vesicles α-synuclein does not distribute randomly but binds only to a fraction of all available vesicles. Furthermore, α-synuclein binding to a supported lipid bilayer observed with total internal reflection fluorescence microscopy displays a much steeper dependence of bound protein on total protein concentration than expected for independent binding. The same phenomenon was observed in the case of α-synuclein binding to unilamellar vesicles of sizes in the nm and μm range as well as to flat supported lipid bilayers, ruling out that nonuniform binding of the protein is governed by differences in membrane curvature. Positive cooperativity of α-synuclein binding to lipid membranes means that the affinity of the protein to a membrane is higher where there is already protein bound compared to a bare membrane. The phenomenon described in this work may have implications for α-synuclein function in synaptic transmission and other membrane remodeling events.

## Introduction

Allostery was discovered in 1961 by Jaques Monod, who referred to the phenomenon as the second secret of life.^1^ Homotropic allostery, or cooperativity, underlies a wide range of biological phenomena, such as transport, cellular signaling, and substrate-activation of enzymes catalyzing committed steps in metabolic pathways. Positive cooperativity enables swift regulation and transitions from free to bound states over a narrow interval of free ligand concentration. Well-known proteins displaying positive cooperativity of binding of ligands or substrates are hemoglobin, calmodulin, and aspartate-transcarbamoylase, involved in oxygen transport, calcium signaling, and nucleotide synthesis, respectively. The data described in the current work reveal positive cooperativity of α-synuclein binding to phospholipid membranes.

α-Synuclein is an intrinsically disordered protein of 140 amino acid residues, which *in vivo* is found predominantly in neurons at presynaptic termini. The concentration of α-synuclein in cells has been estimated to be around 20 μM, and the local concentration in neuronal synapses reaches 50 μM.^2^ The protein is known for its aberrant aggregation associated with a number of neurodegenerative disorders, including Parkinson’s disease and Lewy-body dementia. Both the function and dysfunction of α-synuclein are associated with its interactions with lipid membranes.^3^

A distinct feature of α-synuclein is its highly asymmetric distribution of hydrophobic as well as negatively and positively charged residues within the polypeptide chain. The protein consists of a 60-amino acid N-terminal region rich in positively charged residues, a central hydrophobic region known as non-amyloid β component (NAC) spanning residues 61–95, and a highly acidic C-terminus (residues 98–140). The sequence contains seven imperfect 11-residue repeats analogous to those found in apolipoproteins, which mediate membrane binding.^4^ α-Synuclein is mainly populating random coil conformations in solution; however, part or all of its first 98 residues adopt an amphipathic α-helix upon association with anionic lipid membranes,^4^ SDS-micelles,^5^ or air–water interface.^6^ In the presence of membranes, the number of residues that adopt an α-helical conformation depends on the proportion between the amount of protein and the available lipid membrane surface area.^7^ Under conditions where there is an excess of lipid membrane surface area, all of the 98 residues form an α-helix.^8^ The 42-residue C-terminal tail remains unstructured in the bound protein but may undergo transient interactions with the membrane.^9^

In conditions of α-synuclein excess over the lipid membrane surface area, where there are significant populations of both free and bound protein, aggregation to amyloid fibrils may take place. On the other hand, in conditions of membrane excess, α-synuclein aggregation is inhibited.^10^ In this work, we study the distribution of α-synuclein over the membrane surface area in both regimes, focusing on the conditions of membrane excess, using confocal microscopy, total internal reflection fluorescence (TIRF) microscopy, fluorescence cross-correlation spectroscopy (FCCS), and cryogenic transmission electron microscopy (*cryo*-TEM). Confocal, FCCS, and *cryo*-TEM results show that in conditions of vesicle excess, α-synuclein does not distribute uniformly, but binds only to a fraction of all available vesicles. A binding curve based on the TIRF images of α-synuclein bound to a supported lipid bilayer shows a steep dependence of bound versus total protein concentration. These findings imply that the affinity of α-synuclein to lipid membranes is much higher in the vicinity of already bound protein molecules as compared to a bare membrane. The experimental observations were modeled using the Adair equation and can be described by a reasonable free energy coupling between binding events (around −10 kJ/mol). The strong positive cooperativity of α-synuclein binding to membranes may be relevant to the healthy function of the protein in membrane remodeling.

## Results and Discussion

### Direct Observation of Nonrandom Distribution of α-Synuclein in a Population of GUVs

**Figure 1:**
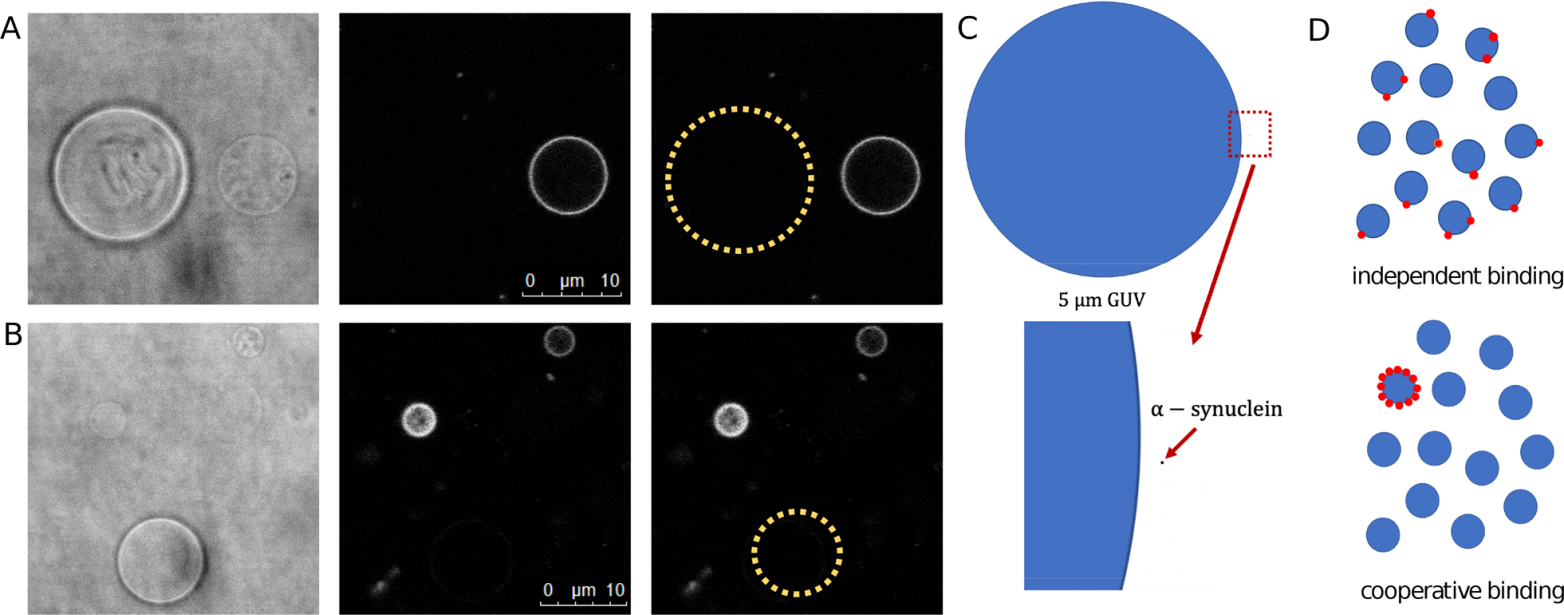
α-Synuclein binding to GUVs. (A, B) Two examples of bright field (left panels) and fluorescence (middle and right panels) images of samples containing DOPC:DOPS 7:3 GUVs and α-synuclein-647, with the protein below the saturation concentration, corresponding to the excess of membrane surface. In the right panels, GUVs missing from the fluorescence images are indicated with yellow dashed circles. (C) Size comparison of a GUV of 5 μm diameter and unfolded α-synuclein (approximated to a dot of 5 nm in radius) showing that for α-synuclein, the membrane of a GUV appears completely flat. (D) Cartoon showing the distribution of protein molecules (red) in a population of vesicles (blue) for the cases of independent binding (left) and fully cooperative binding (right).

When fluorescently labeled α-synuclein is added to lipid membranes in the form of giant unilamellar vesicles (GUVs), the protein distributes in a nonrandom way, as observed with a confocal microscope (Figure 1). We prepared nonlabeled GUVs (DOPC:DOPS 7:3) by electroformation and added fluorescently labeled α-synuclein (α-synuclein-647). The samples were imaged in bright-field and fluorescence modes in parallel. A GUV having no protein bound can be observed in the bright-field but not in the fluorescence mode while a GUV with α-synuclein bound can be seen in both modes. α-Synuclein-647 was added in steps until all GUVs visible in the bright-field mode were also visible in the fluorescence mode.

The images obtained at α-synuclein concentrations below the saturation concentration, corresponding to excess membrane surface, revealed that the protein associates only with some of the vesicles. In a given region of the sample, some vesicles can be seen in both fluorescence and bright-field modes, while other vesicles are only seen in the bright-field mode and thus appear protein-free. In Figure 1A,B, bright-field images are presented together with fluorescence images of the same region. Importantly, in all image frames examined, we consider only GUVs present in the same region of the sample cell, which means that the inhomogeneous distribution of α-synuclein in the population of the vesicles cannot be explained by incomplete mixing of protein in the vesicle solution. α-Synuclein-647 was added stepwise up to the point where all GUVs were saturated with protein. At this stage, all GUVs visible in bright-field mode are also visible in fluorescence mode, implying that all vesicles present in the sample are covered with protein, thus ruling out the possibility that some GUVs were not able to associate with the protein.

A key observation from the confocal experiment is that α-synuclein binds to the GUVs in an all-or-none fashion. We observed no GUV that was only half-filled or had patches of bound protein. All vesicles were either completely fluorescent over the entire circumference of the membrane or displayed no fluorescence at all. In order to exclude any influence of inhomogeneous lipid composition on the observed phenomenon, the confocal experiment was also carried out for GUVs containing 100% DOPS (Figure S1). In a one-component lipid system, all of the GUVs have exactly the same composition. In this case the observations were the same as for the DOPC:DOPS 7:3 system. At low α-synuclein concentrations, only some of the GUVs were fluorescent, while at high protein concentrations all vesicles were fluorescent, indicating protein binding.

### Distribution of α-Synuclein in a Population of SUVs Studied with FCCS

Having studied the distribution of α-synuclein in the excess of GUVs qualitatively, we designed a fluorescence correlation spectroscopy (FCS) experiment in order to analyze this phenomenon in a more quantitative manner. A useful extension of FCS is fluorescence cross-correlation spectroscopy (FCCS), by which the diffusion of two components labeled with different fluorescent dyes is detected simultaneously.

In this experiment, the model system consisted of small unilamellar vesicles (SUVs) containing 0.5% of green fluorescent lipid analogue (Oregon Green DHPE) and α-synuclein-647. We performed the experiments on samples in the lipid/protein ratio (*L*/*P*) range 10–2000. For the system studied, at *L*/*P* = 200 the maximum helical signal in a circular dichroism spectrum is achieved (Figure S3). Therefore, in the *L*/*P* range 10–2000 we cover both regimes of excess protein and excess lipid. The α-synuclein-647 concentration was held constant at 250 nM for all samples while the lipid concentration was varied. For each *L*/*P*, a fresh sample was prepared and incubated for 15 min before the measurement to avoid any effects related to slow kinetics of protein redistribution in the sample.

**Figure 2:**
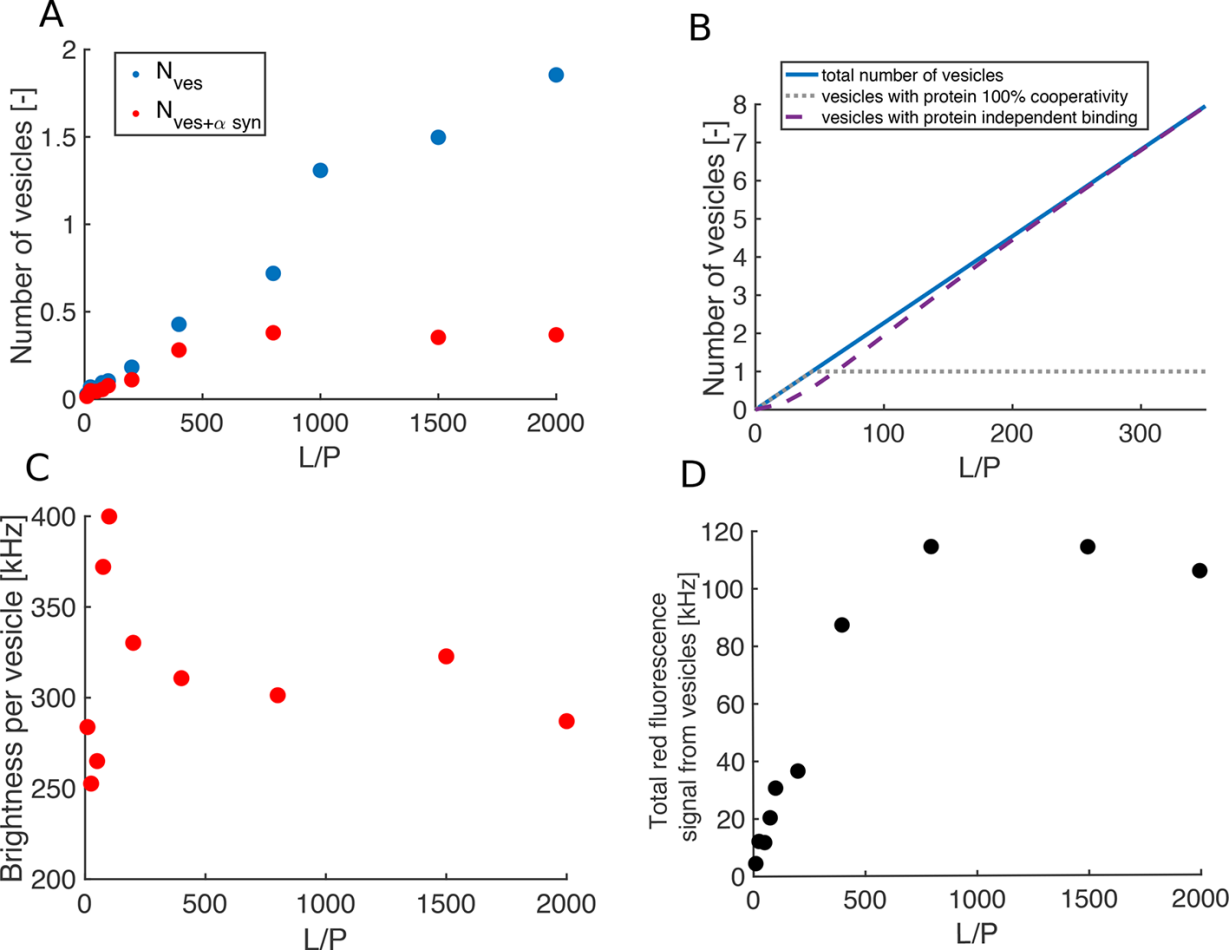
Binding of α-synuclein-647 to DOPC:DOPS 7:3 SUVs with 0.5% Oregon Green DHPE (SUV diameter ≈70 nm). Results from the fluorescence correlation spectroscopy experiment: (A) total number of vesicles *N*_ves_ (blue circles) and the number of vesicles having protein bound *N*_ves+αsyn_ (red circles) extracted from the background-corrected amplitudes of the 488 and 633 nm autocorrelation curves, respectively. (B) Theoretical predictions of the number of vesicles having protein bound as a function of *L*/*P* for the cases of independent (purple dashed line) and infinitely cooperative binding (gray dotted line). The solid blue line corresponds to the total number of vesicles. In the calculations the protein concentration was kept constant while the lipid concentration was varied. Details of the calculations are presented in Experimental Section. (C) Brightness per vesicle in the red channel as a function of *L*/*P*. (D) Total red fluorescence signal from vesicles (i.e., with the α-synuclein signal subtracted) as a function of *L*/*P*. The autocorrelation and cross-correlation curves for free α-synuclein and α-synuclein with SUVs at *L*/*P* of 50, 200, and 2000 are presented in Figure S5.

The amplitude of the autocorrelation curve informs on the number of particles carrying a given fluorophore. When estimating the number of SUVs in the sample, which generate clear intensity spikes on top of the signal from free protein (Figure S4), we treat the signal from free protein as “background” and we perform a background correction of the amplitude of the FCS curve (for details on background correction see Experimental Section). After correction for background fluorescence in the red channel, the number of red particles reports on the number of vesicles decorated with protein (*N*_ves+αsyn_) and can be compared with the number of green particles which reports on the total number of vesicles, *N*_ves_. In Figure 2A, *N*_ves_ and *N*_ves+αsyn_ are plotted as a function of *L*/*P*. We find that as the number of vesicles (*N*_ves_) increases with increasing *L*/*P*, the number of vesicles carrying α-synuclein (*N*_ves+αsyn_) does not increase in proportion. These data can be compared with the theoretical predictions of the number of vesicles with protein bound for independent and cooperative binding plotted in Figure 2B. The calculations were carried out for vesicles with 1000 binding sites. In the case of independent binding, the number of proteins bound to each vesicle follows a binomial distribution (eq 7). In contrast, in the case of infinite cooperativity, the protein occupies the smallest number of the vesicles that it can fill completely regardless of how large excess of vesicles is available. The details of the calculations are presented in Experimental Section.

The brightness per vesicle in the red channel and the total red fluorescence signal from SUVs are plotted in Figure 2C and Figure 2D, respectively. The brightness per vesicle initially increases as a function of *L*/*P* and reaches a maximum at *L*/*P* = 100 which is followed by a decrease, and a plateau is reached at *L*/*P* around 400. The total red fluorescence signal from vesicles is proportional to *N*_ves+αsyn_. Constant brightness per particle and total red fluorescence signal from vesicles in the *L*/*P* range 800–2000 imply no redistribution of α-synuclein-647 molecules on excess vesicles available for binding.

### α-Synuclein Binding to Supported Lipid Bilayer

**Figure 3:**
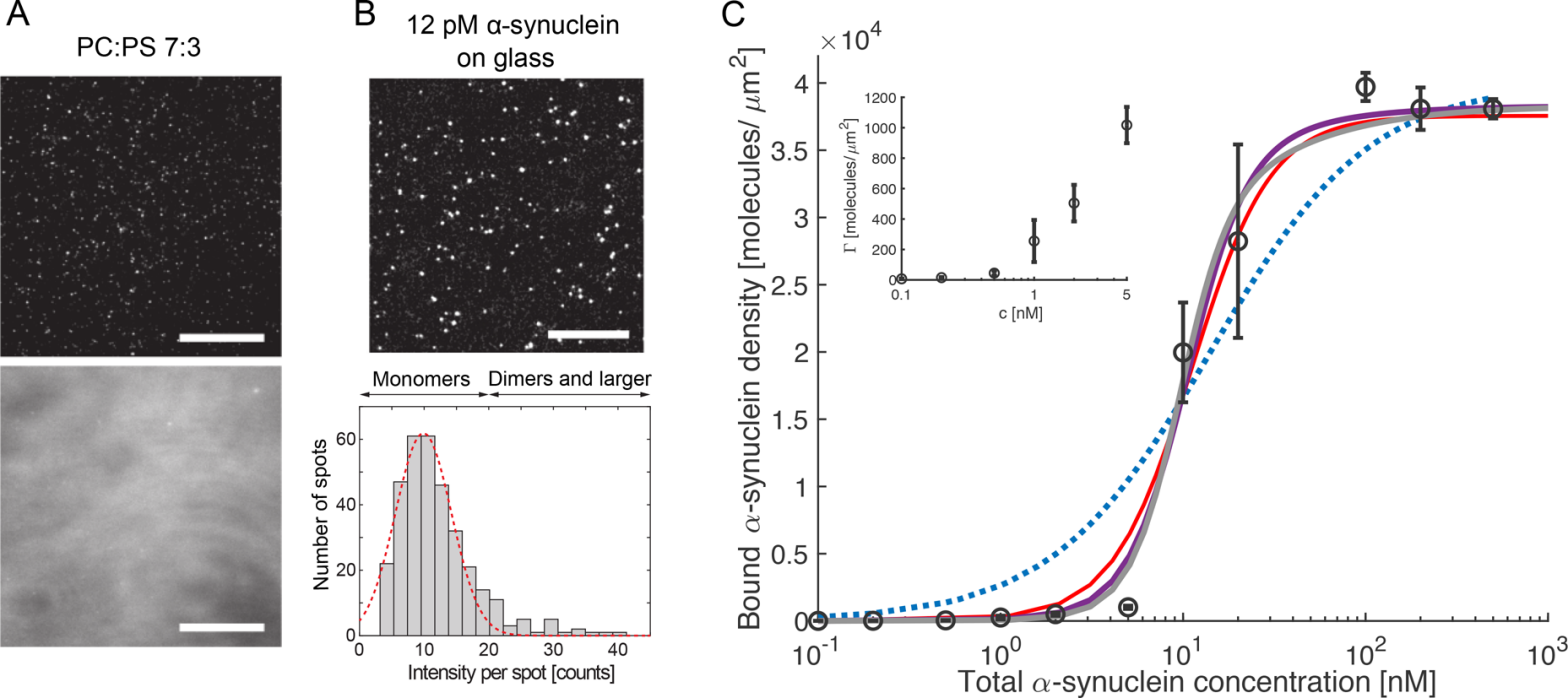
α-Synuclein binding to a flat supported lipid bilayer. (A) Fluorescent signal from a 7:3 POPS:DOPS SLB incubated for 15 min with (top) 0.1 nM α-synuclein and (bottom) 10 nM α-synuclein. (B) (Top) Single molecule fluorescence image of 12 pM α-synuclein adsorbed on a bare glass slide. The scale bar is 20 μm for all images. (Bottom) Histogram showing the total intensity per detected fluorescence ”spot” in the single molecule fluorescence image. The dashed red line is a Gaussian fit to the main peak, showing that 90% of the detected spots exhibit intensity of less than 20 units, corresponding to a monomeric form of the protein (*n* = 335). (C) Density of α-synuclein bound to a SLB for bulk concentrations in the range 0.1–500 nM determined from the fluorescence signal. All data points are the mean ± SE from two or three separate measurements, and the inset shows the first six data points with expanded *y* axis. The dotted blue line shows a fit of the Adair equation (eq 4) for one binding site and corresponds to independent binding. The red, purple, and gray lines represent fits of the Adair equation with two, three, and four coupled binding sites, respectively.

In order to exclude that any curvature effects are responsible for the phenomenon observed in the confocal and FCS experiments, we studied α-synuclein binding to a flat model membrane system-supported lipid bilayer (SLB). POPC:DOPS 7:3 vesicles were deposited on a microscope glass slide to yield an SLB, and α-synuclein-647 was added at total protein concentrations in the range 0.1–500 nM. TIRF images obtained with α-synuclein-647 bulk concentration of 0.1 and 10 nM are shown in Figure 3A. The numbers of α-synuclein molecules bound per μm^2^ for each concentration extracted from the images are plotted in Figure 3C. Already at very low protein concentrations (5 nM and lower) there is binding of α-synuclein to the membrane with a small increase in protein density at the membrane with increasing concentration. There is a sharp increase, by a factor of 20, between 5 and 10 nM, where the density of bound α-synuclein reaches 20 000 molecules/μm^2^. Increasing α-synuclein bulk concentration further leads to a steady-state bound density of 40 000 molecules/μm^2^ which is constant between 100 and 500 nM, pointing toward that the surface of the lipid bilayer is saturated with protein.

In order to make sure that the protein sample that was added to the bilayer is monomeric, a control experiment was performed where α-synuclein-647 binding to a bare glass slide was studied. The intensity of each fluorescent spot was analyzed and plotted in a histogram in Figure 3B (for details on single molecule fluorescence analysis see Experimental Section). On the basis of the intensity histogram, α-synuclein was found to be at least 90% monomeric, whereas the rest cannot be resolved as individual monomers with the resolution of the technique, meaning that they are bound to the surface at a distance of 200 nm or less. There were no signs of larger aggregates, and the higher intensity dots may also indicate a small fraction of dimers, trimers, or tetramers.

The binding curve (Figure 3C) was fitted to the Adair equation (eq 4) assuming *N* = 1 or 2, 3, 4, etc. coupled binding sites. The case with one binding site (blue dotted line in Figure 3C), corresponding to independent binding, does not fit the experimental data. The equation for two coupled binding sites fits the data with infinite cooperativity (free energy coupling between binding events, ΔΔ*G*, being less than or equal to −45 kJ/mol). The Adair equation for three and more binding sites fits the data even better and gives a reasonable ΔΔ*G* (−12 and −8 kJ/mol for three and four coupled binding sites, respectively).

### Visualization of α-Synuclein Distribution in a Population of SUVs Using *Cryo*-TEM

**Figure 4:**
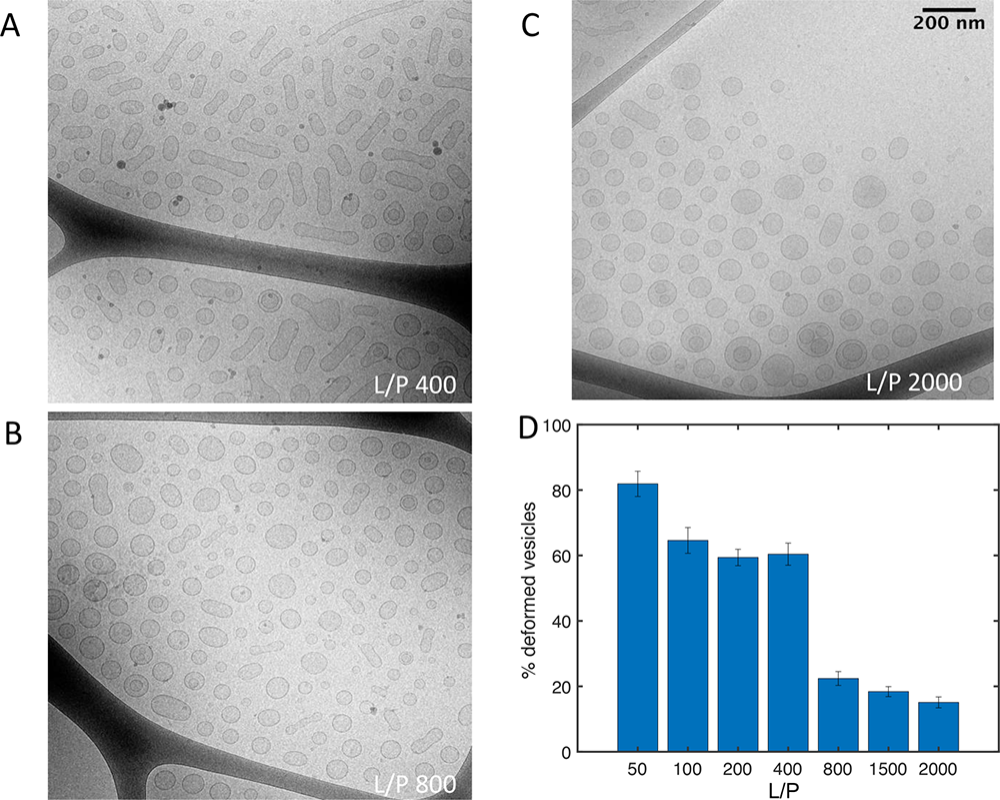
α-Synuclein binding to SUVs studied with *cryo*-TEM. (A–C) Examples of *cryo*-TEM images of small unilamellar vesicles (SUVs) with α-synuclein at different lipid/protein ratios. The images of samples at *L*/*P* of 50, 100, 200, and 1500 are presented in Figure S7. The scale bar is the same as in (C) for all images. (D) Percentage of deformed vesicles (mean ± SD) calculated from six different images of each sample from one experiment assuming no fusion of vesicles. The number of analyzed vesicles was 5330.

The binding of α-synuclein to lipid vesicles was also studied using *cryo*-TEM, a technique that does not rely on fluorescent probes. Here, we took advantage of the observation that SUVs undergo deformation upon α-synuclein binding. In the absence of α-synuclein, the SUVs are almost perfectly spherical in shape (Figure S6). Upon the addition of α-synuclein, there is a clear deformation of the vesicles (Figure 4 and Figure S7), which is consistent with previous reports for similar systems.^7,11,12^ Our interpretation of the *cryo*-TEM images is that deformed vesicles have protein bound, while the spherical ones are protein-free. The fraction of deformed SUVs assuming no vesicle fusion was estimated for *L*/*P* ratios in the range 50–2000 and illustrated in Figure 4D. At high *L*/*P*, 1500 and 2000, less than one-fifth of all vesicles are deformed. When going from *L*/*P* 800 to 50, this fraction increases sharply to around 80% at *L*/*P* 50 corresponding to a situation where almost all vesicles are deformed and thus appear to carry protein.

The analysis results of the fractions of deformed SUVs under the less likely assumption that vesicles undergo α-synuclein induced fusion are presented in Figure S9. For more details on the analysis, see Supporting Information. Regardless of the assumptions of the analysis, the results are quantitatively the same and lead to the same conclusion.

### Cooperative Binding to GUVs and Alternative Explanations

In the confocal experiment we observed that α-synuclein localizes only to a subset of all vesicles at total protein concentrations below saturation concentration and that the protein binds to GUVs in an all-or-none fashion. This led us to formulate the hypothesis that α-synuclein binding to lipid membranes is characterized by strong positive cooperativity.

It is well-known that α-synuclein has a much higher affinity for membranes that contain anionic lipids as compared to purely zwitterionic membranes.^4,13^ We showed however that nonrandom distribution of protein could not be attributed to inhomogeneous distribution of the lipid species between the vesicles (in a DOPC:DOPS 7:3 system) as the same behavior was observed in a system containing only one lipid species (100% DOPS system) (Figure S1).

The GUV sample is rather polydisperse with a wide range of vesicle sizes. One could therefore argue that the differences in membrane curvature between the different vesicles could impact protein binding. There are numerous reports in the literature suggesting that α-synuclein binds to small vesicles with higher affinity than to larger vesicles, and this effect has in some cases been attributed to the differences in membrane curvature.^13−15^ Those studies used small vesicles with a diameter of several tens or hundreds of nm, in which cases, at least for the smallest vesicles, curvature effects may be relevant given the size of the protein. However, this cannot explain the observations of inhomogeneous binding to GUVs as the diameter of these vesicles is in the μm range. For a protein of α-synuclein size (radius of gyration of 3–4 nm ^16^ and length of its full helix when bound of 15 nm^17^), the GUV membranes appear completely flat as illustrated in scale in the cartoon in Figure 1C. The size of the GUV is thus far above any possible curvature-sensing limit for this protein. In the confocal experiment, we observed, however, that in conditions of vesicle excess, the smallest of the GUVs (a few micrometers in diameter) are filled with protein first. For a smaller vesicle, less protein is needed to completely fill the membrane surface as compared to a larger vesicle. Therefore, the fact that in conditions where there is an excess of GUVs, only the smaller ones appear to have protein bound is a manifestation of cooperativity (see Theoretical Predictions of Protein Distrubution in a Population of Vesicles and Figure S2). We emphasize that a size difference between vesicles is not a prerequisite for cooperative binding to occur, which is also confirmed in the FCCS and *cryo*-TEM studies of α-synuclein binding to vesicles with a narrow size distribution. Finally, we point out that an ultimate argument against membrane curvature being responsible for the observed phenomenon is that we observed strongly cooperative binding of α-synuclein also to a flat supported lipid bilayer where the curvature is clearly zero (Figure 3C).

### Quantification of α-Synuclein Distribution on Membranes as a Function of *L*/*P* Ratioαα

In the FCCS and *cryo*-TEM experiments, we studied α-synuclein binding to a population of highly monodisperse SUVs in contrast to a polydisperse GUV sample. The results of the FCCS experiment provide information on the numbers of particles labeled with different fluorophores in the focal volume. The numbers of green and red particles, corresponding to the total number of vesicles and the number of vesicles decorated with protein, respectively, show that in conditions of vesicle excess, the total number of vesicles (*N*_ves_) is higher than the number of vesicles carrying protein (*N*_ves+αsyn_) (Figure 2A). This indicates that some of the vesicles in the sample have no α-synuclein bound. The theoretical predictions of the protein distribution in a population of vesicles show that in the case of independent binding, at high *L*/*P* (corresponding to vesicle excess) the protein distributes over all of the available vesicles (Figure 2B). Therefore, on the basis of the results in Figure 2A, α-synuclein binding to vesicles cannot be described as independent but as cooperative. Importantly, in the FCCS experiment both the total red fluorescence signal from vesicles and brightness per vesicle in the red channel stay constant in the *L*/*P* range 800–2000. This indicates that in this range α-synuclein molecules remain bound only to a constant number of the available vesicles.

While the confocal, TIRF, and FCCS experiments employed α-synuclein linked to a relatively large fluorescent probe, the size of which corresponds to ∼10 amino acid residues, *cryo*-TEM allowed us to study the association of a nonlabeled protein with nonlabeled lipid membranes. The analysis of *cryo*-TEM images of samples at different *L*/*P* ratios reveals that in conditions of vesicle excess, only a fraction of all vesicles (around 20%) has protein bound.

### Theoretical Predictions of Protein Distribution in a Population of Vesicles

The nonrandom distribution of α-synuclein on the available membrane surface area, as observed in the confocal, FCCS, and *cryo*-TEM experiments, can be understood on the basis of the predicted outcomes for independent and cooperative binding. This can be described using the so-called Adair equation,^18^
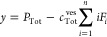
1where *y* is the free protein concentration, *P*_Tot_ is the total protein concentration, *c*_Tot_^ves^ is the total vesicle concentration, *n* is the number of protein-binding sites per vesicle, and
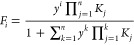
2the fraction of vesicles at each intermediate occupancy *i*. Thus, the fraction of vesicles with no protein bound is
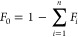
3

Equation 2 used as an input the macroscopic binding constants (*K*_*j*_), which do not refer to any specific binding sites but relate to the first bound protein per vesicle (*K*_1_), the second bound protein per vesicle (*K*_2_), and so on. We solved this equation using a Newton–Raphson method for a range of combinations of *P*_Tot_ and *c*_Tot_^ves^ for the cases of vesicles with *n* = 2 and *n* = 10 binding sites.

**Figure 5:**
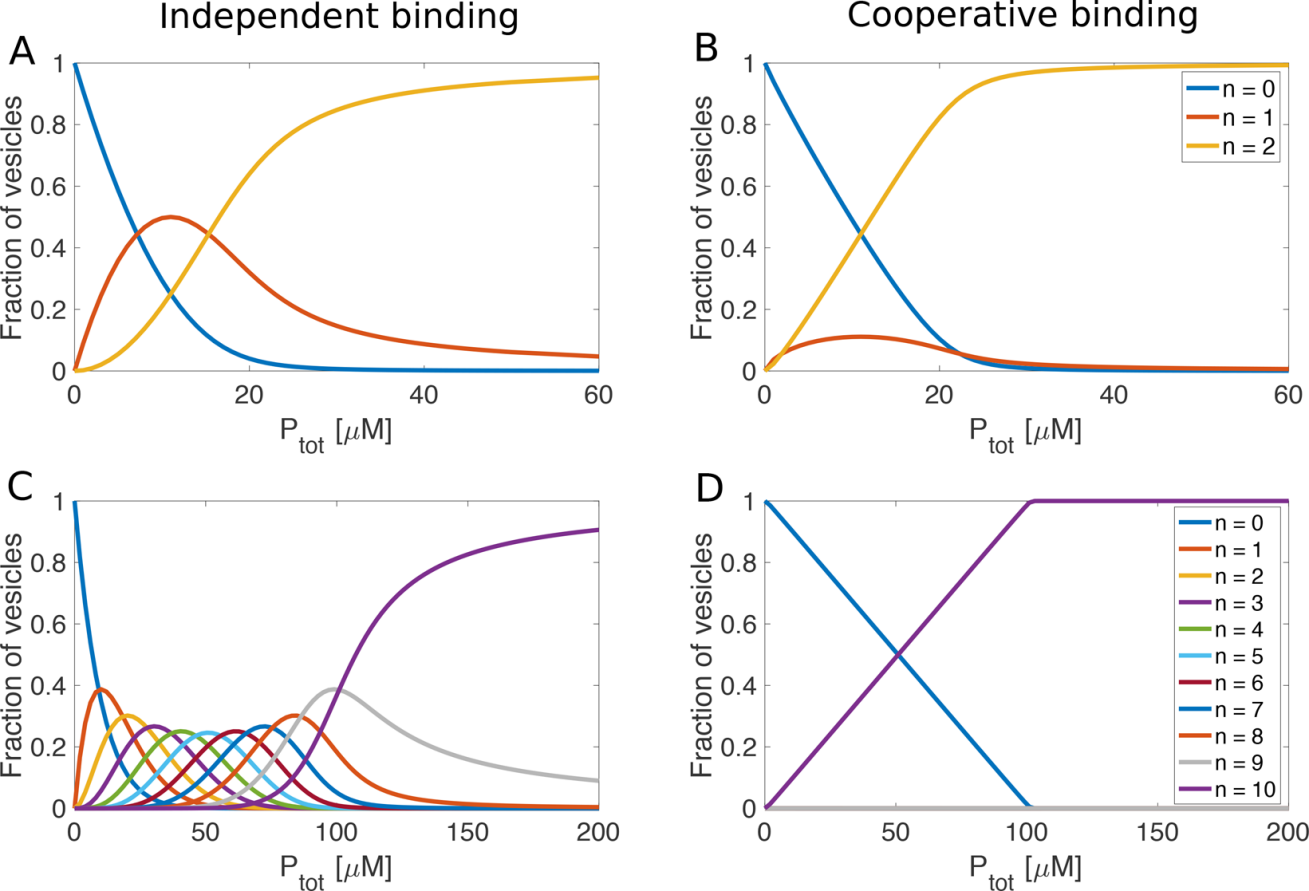
Calculations using the Adair equation: fractions of hypothetical vesicles with *n* proteins bound as a function of total protein concentration for vesicles with 2 (upper panels) and 10 binding sites (lower panels) for the cases of no cooperativity (left) and strong positive cooperativity (right). Values of the macroscopic binding constants for the case of independent binding were calculated from *K*_*j*_ =  × 10^6^ M^–1^ where *N* is the total number of binding sites. Values of the macroscopic binding constants for positively cooperative binding to vesicles with two binding sites were *K*_1_ =  × 10^6^ M^–1^ and *K*_2_ = 4 × 10^6^ M^–1^, assuming an average affinity of 1 × 10^6^ M^-1^ and a free energy coupling of ΔΔG=-10 kJ mol^-1^. For the case of vesicles with 10 binding sites and cooperative binding, the macroscopic binding constants were K_1_ = 0.0745 M^-1^, K_2_ = 2.15 M^-1^, K_3_ = 81.4 M^-1^, K_4_ = 3400 M^-1^, K_5_ = 1.5 × 10^5^ M^-1^, K_6_ = 6.7 × 10^6^ M^-1^, K_7_ = 2.9 × 10^8^ M^-1^, K_8_ = 1.2 × 10^10^ M^-1^, K_9_ = 4.7 × 10^11^ M^-1^ and K_10_ = 1.3 × 10^13^ M^-1^, assuming an average affinity of 1 × 10^6^ M^-1^ and a free energy coupling of ΔΔG=-10 kJ mol^-1^.

We assumed the same average affinity for the cases of independent and cooperative binding; i.e., the product of all macroscopic binding constants was the same in both cases. In the case of independent binding to a population of vesicles with two binding sites of identical affinities, the macroscopic binding constants are *K*_1_ = 2 × 10^6^ M^–1^ and *K*_2_ =  × 10^6^ M^–1^. To model the case of very high cooperativity, we used *K*_1_ =  × 10^6^ M^–1^ and *K*_2_ = 4 × 10^6^ M^–1^, corresponding to a 64-fold increase in affinity per binding step with −10 kJ/mol free energy coupling between the binding events. The values of the macroscopic binding constants for the case of vesicles with 10 binding sites are reported in the legend of Figure 5. The free energy coupling between binding events is −10 kJ/mol. In these calculations we kept the vesicle concentration constant at 10 μM and varied the protein concentration from 0 to 60 μM for vesicles with *n* = 2 binding sites and from 0 to 200 μM for vesicles with *n* = 10 binding sites.

The change in the calculated values of the fractions of empty and completely filled vesicles as well as all the intermediate states with increasing protein concentration can be compared with the observations from the confocal experiment where α-synuclein was added stepwise to a solution containing GUVs (Figure 1). Clearly, in the absence of cooperativity (Figure 5A,C), intermediate states are significantly populated during the titration, but in the case of positive cooperativity (Figure 5B,D) the solution is at all stages dominated by the fully free and completely filled vesicles. Although vesicles of any possible size can accommodate much more than 10 α-synuclein molecules on their surface, it is sufficient to use 10 coupled sites and a reasonable level of cooperativity (−10 kJ/mol) to fully suppress the populations of all intermediate states.

The Adair equation was also solved for an equimolar mixture of vesicles with 2 and 10 binding sites. The plots of fractional saturation versus total protein concentration are shown in Figure S2. In the case of cooperative binding, at low protein concentration, the small vesicles (with 2 binding sites) are preferentially filled, because the first binding constant of 2 is higher than the first binding constant of 10, under the assumption of equal average affinity and the same level of cooperativity per step. Above a fractional saturation of 0.5 the trend reverses. This is consistent with the experimental observations in the confocal experiment, where at α-synuclein concentration below saturation, the smaller GUVs were filled with protein first.

### Cooperativity and Exchange

The observations presented here indicate that the exchange between free and membrane-associated α-synuclein is relatively rapid. The establishment of a nonrandom distribution during the experimental dead-times (on the order of seconds) is compatible with multiple on–off events for each α-synuclein molecule during this time, thus allowing the protein molecules to find the energetically most favorable distribution. The off rates of proteins from a variety of binding partners, including other macromolecules, small ligands, and surfaces, vary widely from below 10^–6^ to 10^5^ s^–1^. The dissociation rate constants for protein–protein complexes are typically found in the lower end of this range^19−21^ and for protein–ligand complexes in the higher end.^22^ The relatively high exchange rate inferred for α-synuclein may be explained by its character of a peripheral membrane protein interacting only with the outermost part of the bilayer (the outer headgroup and upper acyl layer).^23,24^

### Molecular Origin of the Cooperativity

While our data provide no information on the molecular origin of the cooperativity, we may speculate on possible causes. The more favorable binding of α-synuclein next to other α-synuclein molecules on the membrane, compared to a bare membrane, must be related to the balance between the lipid–lipid, protein–lipid, or protein–protein interactions. It is possible that when α-synuclein adsorbs and folds on the membrane, it also creates a new hydrophobic interface along the α-helix or in the bilayer. The free energy of binding of the second α-helical protein may be lower for that hydrophobic location as compared to the bare membrane surface. This may then lead to binding being more favorable for clusters as compared to isolated binding. It is also possible that unfavorable effects on the lipids such as reduced lateral diffusion and thereby a decrease in entropy may to a higher degree be “paid” by the first protein bound in a given spot. Alternatively, the positive cooperativity may have its origin in the interactions of the protein or membrane components with water and counterions, with the net desolvation being more favorable (or less unfavorable) for cooperative compared to isolated protein binding.

### Indications of α-Synuclein Binding Cooperativity in the Literature

α-Synuclein binding to lipid membranes has been studied extensively for more than two decades using many different biophysical techniques. Still, it has not been characterized as cooperative before. In part this is due to the use of “bulk” experimental techniques that report (directly or indirectly) on the total fractions of free and bound protein rather than the protein’s distribution over individual vesicles.^4,7−9,24,25^ α-Synuclein binding has also been characterized using single-molecule techniques such as FCS;^13,26^ however, the data were used to extract the bound protein concentrations as a function of lipid concentrations, thus again reporting on a “bulk” property of the system. The current experiments are based on established techniques but were designed to reveal α-synuclein binding to individual vesicles (GUVs in confocal microscopy, SUVs in FCS and *cryo*-TEM). Key aspects were the use of the bright-field mode to observe the nonfluorescent vesicles in the confocal experiment and the use of membrane excess conditions to enable coexistence of protein-free and protein-bound vesicles.

Despite the fact that α-synuclein binding to membranes has not been described as cooperative before, there are numerous reports in the literature that corroborate our findings. In 2008, Lee et al.^27^ showed that α-synuclein localizes only to a subset of the vesicles while other synaptic proteins (synaptophysin and synaptobrevin) were found in all analyzed fractions of synaptic vesicles from rat brain homogenate. The authors suggested that such specific localization of the protein may be linked to its normal function in synaptic transmission. Bureé et al.^3^ used cross-linking and FRET to show that α-synuclein molecules assemble into higher order structures on the surface of vesicles. Protein molecules bound to vesicles could be cross-linked into groups of 8 and more while no cross-linking was observed in absence of vesicles, thus indicating clustering of α-synuclein molecules on the membrane surface.

Nuscher et al.^28^ studied binding of α-synuclein to small unilamellar vesicles using isothermal titration calorimetry. Titration of SUVs into a solution of α-synuclein was accompanied by an exothermic enthalpy change up to *L*/*P* 300. Further addition of SUVs up until *L*/*P* 900 did not result in any heat effect apart from the heat of dilution of vesicles. This result suggests that adding more SUVs above the point where all α-synuclein molecules are bound to the vesicles does not result in redistribution of the protein which would likely be accompanied by a heat effect due to loss of protein–protein interactions. Drescher et al.,^29^ using double electron–electron resonance (DEER), showed that α-synuclein forms “supramolecular well-ordered arrays with well-defined molecular contacts”. In this study single-cysteine mutants of α-synuclein (Cys introduced at positions 9,18, 69, and 90) were labeled with a probe containing an unpaired electron. DEER experiments revealed distinct distances between the pairs of spins of α-synuclein molecules bound to vesicles as opposed to a homogeneous distribution of spins characteristic for a monomeric protein in solution. The distances measured depend on the position of the unpaired electron in the polypeptide chain. On the basis of these data, two models of dimers of α-synuclein with a broken helix in a horseshoe conformation were proposed as the simplest building blocks of the supramolecular structure. Importantly, the distance distributions measured were not affected by changing the *L*/*P* from 250 to 1000, which would be the case if the supramolecular structures formed by proteins at lower *L*/*P* would be diluted by adding more vesicles. This again shows that α-synuclein molecules do not distribute uniformly over the accessible membrane area. The antiparallel arrangement of the helices of the α-synuclein molecules in the dimer, which emerged from the DEER data, is consistent with Bureé et al.^3^ FRET results discussed above.

## Concluding Remarks

Our results reveal that α-synuclein binding to lipid membranes occurs with strong positive cooperativity. We have shown this for flat supported lipid bilayers and unilamellar vesicles of different sizes (SUVs in the nm range and GUVs in the μm range). We ruled out the possibility that the observed phenomenon could be due to differences in membrane composition or curvature. A reasonable free energy coupling between the binding events (around −10 kJ/mol) is sufficient to explain the observed phenomenon. We emphasize that the conditions of excess of membranes were necessary to show the phenomenon experimentally but are not a prerequisite for it to occur. On a synaptic vesicle densely packed with proteins,^30^ the binding cooperativity would be manifested by α-synuclein molecules binding in patches instead of distributing uniformly. Finally, we argue that the cooperativity of α-synuclein binding to membranes is very likely related to its function in membrane remodeling and synaptic vesicle trafficking, processes that would be well controlled by a protein that segregates into distinct patches on the membrane.

## Experimental Section

### α-Synuclein Expression and Purification

α-Synuclein of human wild-type sequence, or with a N122C mutation, was expressed in *E. coli* from Pet3a plasmids with *E. coli*-optimized codons (purchased from Genscript, Piscataway, New Jersey). The wild-type protein was purified using heat treatment and ion-exchange and gel filtration chromatography, as previously described.^31^ The N122C mutant was purified using the same protocol but with 1 mM dithiothreitol (DTT) included in all buffers. Each purified protein was stored as multiple identical aliquots at −20 °C. All experiments started with gel filtration of such aliquots on a 10 × 300 mm Superdex 75 column (GE Healthcare) to isolate fresh monomer in 10 mM MES buffer at pH 5.5. All measurements were carried out under these buffer conditions.

### α-Synuclein Labeling

α-Synuclein N122C mutant was labeled with Alexa Fluor 647 maleimide. Gel filtration on a 10 mm × 300 mm Superdex 75 column was used to remove DTT from the protein and to exchange the buffer to 20 mM sodium phosphate, pH 8.0. One molar equivalent of Alexa Fluor 647 maleimide dye was added from a 5 mM stock in DMSO to the protein solution, which was incubated for 1 h at room temperature in the dark. Excess free dye and phosphate buffer were removed using gel filtration on a 10 mm × 300 mm Superdex 75 column in 10 mM MES, pH 5.5. In the text, the Alexa Fluor 647 labeled α-synuclein is referred to as α-synuclein-647.

### Lipids

Lyophilized lipids: 1,2-dioleoyl-*sn*-glycero-3-phospho-l-serine sodium salt (DOPS), 1,2-dioleoyl-*sn*-glycero-3-phosphocholine (DOPC), 1-palmitoyl-2-oleoyl-*sn*-glycero-3-phosphocholine (POPC), Oregon Green 488 1,2-dihexadecanoyl-*sn*-glycero-3-phosphoethanolamine (DHPE-488), and 1,2-distearyl-*sn*-glycero-3-phosphoethanolamine-N-(TopFluor AF488) ammonium salt (AF488-PE) were purchased from Avanti Polar Lipids (Alabaster AL).

### GUV, SUV, and SLB Preparations

Giant unilamellar vesicles (GUVs) were prepared using electroformation. 15 μL of 2 mg/mL 7:3 (molar ratio) DOPC:DOPS mixture or 100% DOPS in chloroform:methanol (7:3 volume ratio) was deposited on the conductive side of an indium tin oxide covered glass slide and left in a vacuum oven for the solvent to evaporate for 24 h. The coverslip was mounted on a bottomless 6-channel slide with a self-adhesive underside (Ibidi, GmbH). The lipid layer was then hydrated with 120 μL of buffer (10 mM MES, pH 5.5) through the channel. The electroformation was carried out for 2 h. Alternating current was created using alternating voltage, 30 V, at a frequency of 50 Hz for the synthesis of DOPC:DOPS system and 1 kHz for the pure DOPS system.

Small unilamellar vesicles (SUVs) were prepared by extrusion using Avanti Mini Extruder (Avanti Polar Lipids). The desired volume of 7:3 (molar ratio) DOPC:DOPS mixture in chloroform:methanol (7:3 volume ratio) was left overnight in a vacuum oven at room temperature for the solvent to evaporate. The dried lipids were then hydrated with 10 mM MES buffer at pH 5.5 and left on stirring for 2 h at room temperature. Highly monodisperse SUVs were obtained by extruding 21 times through 50 nm pore size filters that had been saturated with the same lipids before use. The size distribution and polydispersity index were analyzed using Malvern Zetasizer Nano-Z (Malvern Instruments Ltd.). The average hydrodynamic radius of SUVs was 37 nm and polydispersity index 0.06. SUVs used for FCS experiment were prepared with 0.5% Oregon Green 488 DHPE (DHPE-488).

Vesicles for SLB preparation composed of 7:3 (molar ratio) POPC:DOPS with the addition of 0.1 wt % of the labeled lipid AF488-PE were prepared by dissolving the required amount of lipids in chloroform and dried under a gentle N_2_ stream. A lipid film was obtained and then hydrated in HBS buffer (10 mM HEPES (2-[4-(2-hydroxyethyl)piperazin-1-yl]ethanesulfonic acid), 150 mM NaCl, pH 7.4). The mixture was vortexed six times, yielding a slightly milky dispersion which was incubated on ice. After 1 h, the dispersion was sonicated using a tip sonicator (CV18 model, Chemical Instruments AB) set at 40% amplitude in pulse mode (10 s on followed by 10 s off) for 15 min of total sonication time. The vesicles samples were stored at 4 °C prior to use. A 0.15 mm thick round glass slide (number one coverslips ⌀ 25 mm, Thermo Fisher Scientific) was cleaned at 80 °C for 30 min in piranha solution (3:1 v/v of 99% H_2_SO_4_ and 30% H_2_O_2_, both from Sigma) and then rinsed excessively in running, distilled water. A press-to-seal silicon well (silicon isolators, 12 × 4.5 mm diameter, 1.7 mm depth, Grace Biolabs) was attached to the clean glass slide. The vesicles were diluted 1:10 (v/v) in HBS buffer and incubated for 1 h at room temperature in the well. After 1 h of incubation in the well at room temperature and in dark conditions, the excess vesicles were removed from the formed SLB by washing at least five times first with HBS buffer and then with the 10 mM MES at pH 5.5 buffer used for the α-synuclein binding experiments.

### Confocal Laser Scanning Microscopy

Confocal micrographs were acquired on an inverted confocal laser scanning microscope Leica DMI6000 with an SP5 tandem scanner operating in resonant mode. A 100× (1.4 NA) oil immersion objective was used. The GUVs were imaged in the 6-channel slide used for preparation. A 0.3 μM solution containing α-synuclein-647 and nonlabeled wild-type at 1:20 molar ratio was added in 2 μL steps to the channel.

### TIRF Microscopy

A Nikon Eclipse TE2000-U microscope equipped with a Hamamatsu ORCA-Flash4.0 LT Digital scientific CMOS camera (C1140-22U) and a Nikon Apo TIRF 60× magnification oil-immersion objective was used for the fluorescence measurements. The SLB and the α-synuclein were illuminated by Cobolt MLD compact diode lasers operating at 488 nm (30 mW) and 638 nm (60 mW) for the SLB and α-synuclein, respectively.

The mobility of SLBs used for the TIRF experiments was in all cases evaluated by means of fluorescence recovery after photobleaching (FRAP) where a small area of the SLB was photobleached by focusing the laser illumination to the center of the SLB and studying the recovery after bleaching. A high recovery (>98%) was observed in all cases, which was analyzed by the MATLAB program frap_analysis.^32^ Having ensured a good quality of the SLB, α-synuclein was added to the well at increasing sequential concentrations (0.1, 0.2, 0.5, 1, 2, 5, 10, 20, 100, 200, and 500 nM), each of which was incubated for 15 min prior to imaging. The images of α-synuclein on the SLB’s surface were recorded in three different locations of the lipid bilayer with α-synuclein in the solution (similar results were obtained when imaging after rinsing with buffer). The following ND filters were used when illuminating the protein at the different concentrations: 0.1 to 1 nM (ND0.5), 2 to 5 nM (ND1), 10 to 20 (ND2), 100 to 500 nM (ND3). For the highest protein concentration (500 nM), a FRAP experiment was performed after rinsing the sample, showing that the majority of α-synuclein even at these saturated concentrations was mobile. The SLB and α-synuclein images were acquired with 100 and 60 ms of exposure time, respectively, via μManager version 1.4.^33^ The experiments up to 20 nM were repeated on three separate SLBs, whereas the 100, 200, and 500 nM experiments were repeated twice.

We performed a control experiment where α-synuclein was added to a POPC bilayer. The difference in density of bound α-synuclein between the pure POPC and the 7:3 POPC/DOPS SLB was low at low concentration of proteins (<1 nM), where the images indicate that α-synuclein binds to defects. At higher concentrations (>10 nM), the two signals deviate with a much higher amount (∼140 times) of adsorbed α-synuclein in the presence of PS in the bilayer.

### Single Molecule Experiments and Data Analysis

In order to convert the fluorescence intensity to α-synuclein density, the intensity from a single α-synuclein molecule was determined. For this, a 12 pM solution of α-synuclein in 10 mM MES at pH 5.5 was added to a glass cover slide for 15 min before rinsing with buffer and images of the sample were recorded with a ND0.5 filter. This allowed for single molecules to be visualized as bright “spots” on the SLB. Each spot was detected using a customed-made MATLAB script after which the total intensity from this spot was measured. As described previously by us,^34^ this gives a conversion factor between the pixel intensity to protein density for the bound α-synuclein (for ND0.5 the pixel intensity should be divided by approximately a factor of 5 to give protein density in molecules/μm^2^, which scales with the ND filters used). The bound α-synuclein density (Γ) vs the total concentration of protein (*c*) was fitted with the Adair equation, under the assumption that the amount of the bound protein is negligible such that (c) represents the free protein concentration:
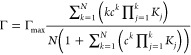
4where Γ_max_ is the maximum density of bound α-synuclein, *N* is the number of coupled binding sites, and *K*_*j*_ are the macroscopic binding constans.

### Fluorescence Correlation Spectroscopy Setup

FCS measurements were performed using a Zeiss 780 confocal laser scanning microscope equipped for FCS and FCCS, with a Zeiss water immersion objective, C-Apochromat 40×/1.2 NA. Samples labeled with Oregon Green were excited at 488 nm and fluorescence emission was collected at 499–622 nm, while Alexa Fluor 647 samples were excited at 633 nm and fluorescence was collected at 641–695 nm. HiLyte 488 (433 μm^2^/s) and HiLyte 647 (320 μm^2^/s)^35^ were used for calibration and yielded τ_Dg_ = 32 μs, ω_g_ = 0.24 μm, and τ_Dr_ = 62 μs, ω_r_ = 0.28 μm, respectively. Thirty FCCS measurements of 10 s duration were carried out in the measurement dish MatTek, 35 mm, 10 mm glass bottom, no. 1.5 glass.

### Fluorescence Correlation Spectroscopy Data Analysis

The concentration and brightness of SUVs carrying α-synuclein would normally be estimated by fitting the red FCS curve to a model with two or three diffusion components, corresponding to SUVs, free α-synuclein-647, and/or free Alexa Fluor 647 dye molecules. However, due to the more than 50-fold higher brightness of the SUVs as compared to single Alexa Fluor 647 molecules, our FCS curves indicated only rarely the presence of any faster component in addition to the SUVs. Instead, the curves showed only a single component, corresponding to the SUVs. The SUV concentration and brightness were therefore estimated by considering the signal from α-synuclein-647 with free dye molecules as background signal. In such a way, the number (*N*) and brightness of particles (CPM) in the red channel correspond only to vesicles with protein but not to free protein or residual free dye.^36^

For the samples where the FCS curve did show a fast component, estimating *N* and CPM by correcting for background yielded almost identical results as analyzing the two components of the FCS curve.

The finding of almost identical results by the two approaches can be understood by comparing their respective equations. In the case when a fast component is visible in the FCS curve, and fitting can be done with a two-component model, the dominating slow component corresponding to SUVs is given by
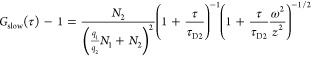
5Here *N*_1_, *q*_1_, *N*_2_, *q*_2_ are the numbers and brightnesses of the fast and the slow components, respectively. In the case when the FCS curve shows only a single component, corresponding to the SUVs, background is corrected for by using
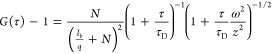
6*I*_b_ is the signal corresponding to background, *N* is the number of vesicles, and *q* is the brightness of a vesicle. We treat the signal from fast molecules as background, i.e., *I*_b_ = *q*_1_*N*_1_, which makes eqs 6 and 5 identical.

Background correction of the green (*A*_g_) and the red (*A*_r_) autocorrelation amplitudes as well as the cross-correlation amplitude (*A*_cc_) was done as follows. From each measurement the intensity histogram of the total detected signal (*I*_t_) was analyzed, where the center position of the main peak, which corresponds to background, was taken as the mean background signal (*I*_b_). *A*_g_ and *A*_r_ were then corrected by multiplication by (*I*_t,*a*_)^2^/(*I*_t,a_ – *I*_b,*a*_)^2^, where index *a* indicates g (green) or r (red), and *A*_cc_ was multiplied by *I*_t,g_*I*_t,r_/((*I*_t,g_ – *I*_b,g_)(*I*_t,r_ – *I*_b,r_)).^37^

### Modeling Fluorescence Correlation Spectroscopy Results

In the FCS experiment we have used SUVs of approximately 70 nm in diameter. Assuming that the lipid-headgroup area in the bilayer is 0.7 nm^2^,^38^ there is around 44 000 lipid molecules in a vesicle (in both the inner and outer leaflets). The surface area occupied by one α-synuclein molecule bound with its full 98 residue fragment is estimated as 15 nm^2^.^17^ Therefore, a vesicle of 70 nm in diameter can accommodate approximately 1000 protein molecules.

The fraction of vesicles carrying protein as a function of *L*/*P* ratio was calculated for the case of independent binding according to the equation
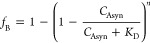
7where *C*_Asyn_ is the total α-synuclein concentration which was held constant at 166 nM (concentration equal to the concentration of binding sites on one vesicle), *K*_D_ is the average dissociation constant per binding event and assumed to be 1 nM, and *n* is the number of binding sites on each vesicle. We assumed *n* = 1000.

In the case of infinite cooperativity, all the protein molecules were assumed to occupy one vesicle regardless of the total number of vesicles.

### Modeling Confocal Microscopy Results Using the Adair Equation for α-Synuclein Distribution in a Population of Equally Sized Vesicles

The macroscopic binding constants used to calculate the fractions of vesicles with 10 binding sites having *n* proteins bound (Figure 5) for a constant vesicle concentration and varying total protein concentration were, for a case of independent binding to a vesicle with 10 binding sites, *K*_1_ = 10, *K*_2_ = 4.5, *K*_3_ = 2.67, *K*_4_ = 1.75, *K*_5_ = 1.2, *K*_6_ = 0.833, *K*_7_ = 0.571, *K*_8_ = 0.375, *K*_9_ = 0.222, and *K*_10_ = 0.1 (×10^6^ M^–1^). Macroscopic binding constants used for a case of cooperative binding were K_1_ = 0.0745 M^-1^, K_2_ = 2.15 M^-1^, K_3_ = 81.4 M^-1^, K_4_ = 3400 M^-1^, K_5_ = 1.5 × 10^5^ M^-1^, K_6_ = 6.7 × 10^6^ M^-1^, K_7_ = 2.9 × 10^8^ M^-1^, K_8_ = 1.2 × 10^10^ M^-1^, K_9_ = 4.7 × 10^11^ M^-1^ and K_10_ = 1.3 × 10^13^ M^-1^, assuming an average affinity of 1 × 10^6^ M^-1^ and a free energy coupling between binding events of ΔΔG=-10 kJ mol^-1^.

### *Cryo*-TEM

α-Synuclein-SUV samples were prepared at different *L*/*P* ratios (50, 100, 200, 400, 800, 1500, 2000). In all samples, the lipid concentration was 20 mM while the α-synuclein concentration was varied accordingly. Specimens for *cryo*-TEM were prepared in an automatic plunge freezer system (Leica EM GP). The climate chamber temperature was kept at 21 °C, and relative humidity was ≥90% to minimize loss of solution during sample preparation. The specimens were prepared by placing 4 μL of solution on glow discharged lacey Formvar carbon coated copper grids (Ted Pella) and blotted with filter paper before being plunged into liquid ethane at −180 °C. This leads to vitrified specimens, avoiding component segmentation and rearrangement, and the formation of water crystals, thereby preserving original microstructures. The vitrified specimens were stored under liquid nitrogen until measured. A Fischione model 2550 cryotransfer tomography holder was used to transfer the specimen into the electron microscope, JEM 2200FS, equipped with an in-column energy filter (Omega filter), which allows zero-loss imaging. The acceleration voltage was 200 kV, and zero-loss images were recorded digitally with a TVIPS F416 camera using SerialEM under low dose conditions with a 10 eV energy selecting slit in place.

## Supporting Information Available

Confocal fluorescence microscopy, total internal reflection fluorescence microscopy, circular dichroism spectroscopy, and cryogenic transmission electron microscopy (PDF)
