# Supplementary material for: Cooperativity of α-Synuclein Binding to Lipid Membranes
Source: ACS Chem Neurosci. 2021 Jun 2;12(12):2099–109. doi: 10.1021/acschemneuro.1c00006 (PMC8291482; doi:10.1021/acschemneuro.1c00006)
Supplement: Supplementary file 1 — cn1c00006_si_001.pdf [file cn1c00006_si_001.pdf]

Supporting Information: Cooperativity of α-synuclein Binding to Lipid Membranes

Katarzyna Makasewicz,*,† Stefan Wennmalm,‡ Björn Stenqvist,† Marco Fornasier,† Alexandra Andersson,† Peter Jönsson,† Sara Linse,¶ and Emma Sparr†

†*Division of Physical Chemistry, Center for Chemistry and Chemical Engineering, Lund University, P.O. Box 124, SE-22100 Lund, Sweden*

‡*Royal Institute of Technology-KTH, Department of Applied Physics, Experimental Biomolecular Physics Group, Scilifelab, 171 65 Solna, Sweden*

¶*Division of Biochemistry and Structural Biology, Lund University, SE-22100 Lund, Sweden*

E-mail: katarzyna.makasewicz@fkem1.lu.se

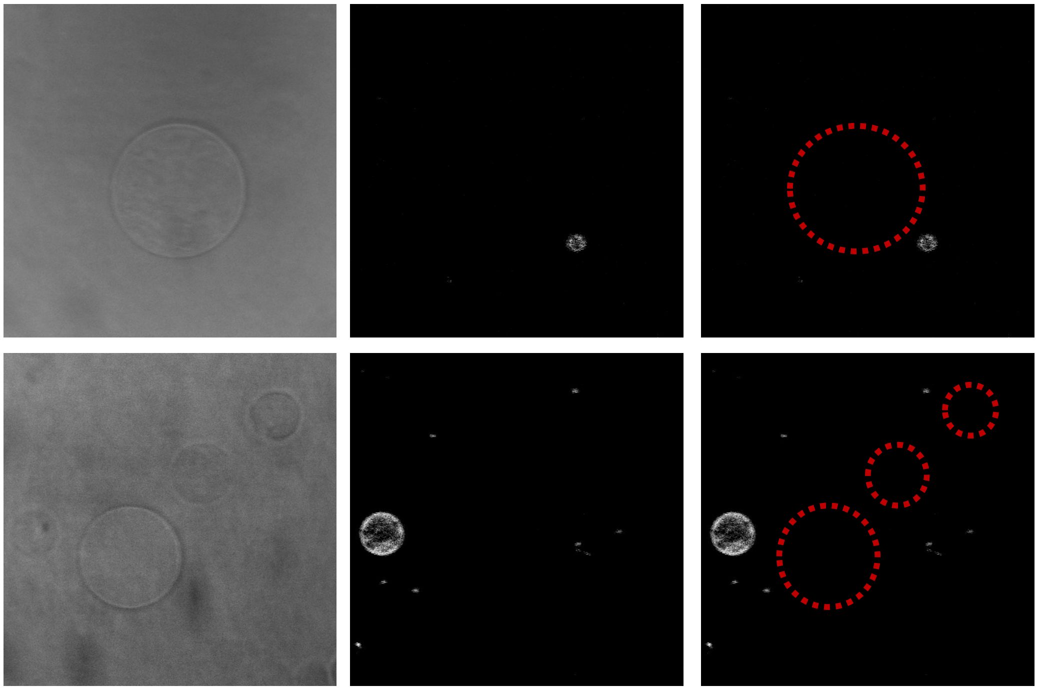

Figure S1: Initial steps of α-synuclein-647 titration of giant unilamellar vesicles (GUVs) prepared by electroformation from 100 % DOPS. Left: bright-field images of GUVs. Middle: fluorescence images of the same GUVs showing only the vesicles having α-synuclein bound. Right: GUVs missing from the fluorescence image were indicated with a red dotted lines.

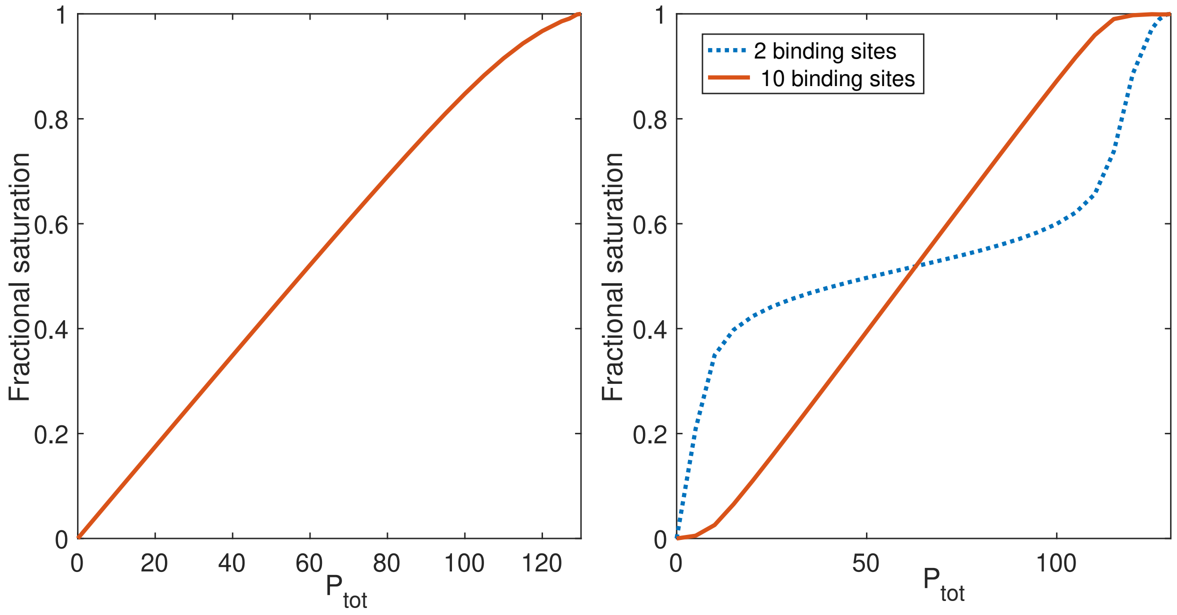

Figure S2: The Adair equation solved for protein binding to an equimolar mixture of vesicles with 2 and 10 binding sites for the cases of independent binding (left) and cooperative binding (right). In the case of independent binding the fractional saturation curves for both vesicle types overlap.

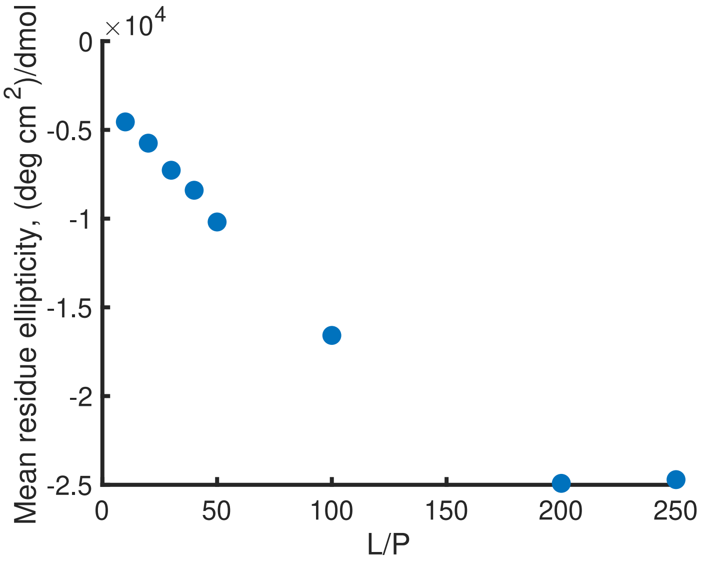

Figure S3: Circular dichroism spectroscopy data: Mean residue ellipticity at 222 nm as a function of lipid/protein (mol) for α-synuclein wild type with DOPC:DOPS 7:3 (mol) SUVs of around 70 nm in diameter.

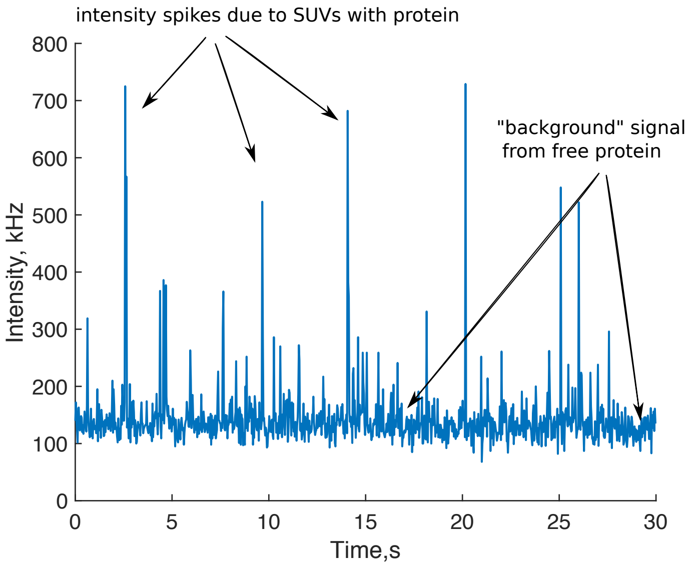

Figure S4: Fluorescence cross-correlation spectroscopy. Intensity trace from the red channel where vesicles carrying α-synuclein and free α-synuclein are detected. The vesicles with protein generate clear intensity spikes on top of the signal from free protein. In order to estimate the number of protein-decorated vesicles in the sample, we treat the signal from free protein as "background" and we perform background-correction of the amplitude of the FCS curve. After correction, the number of red particles reports on the number of vesicles decorated with protein. For more details on background correction see Materials and Methods.

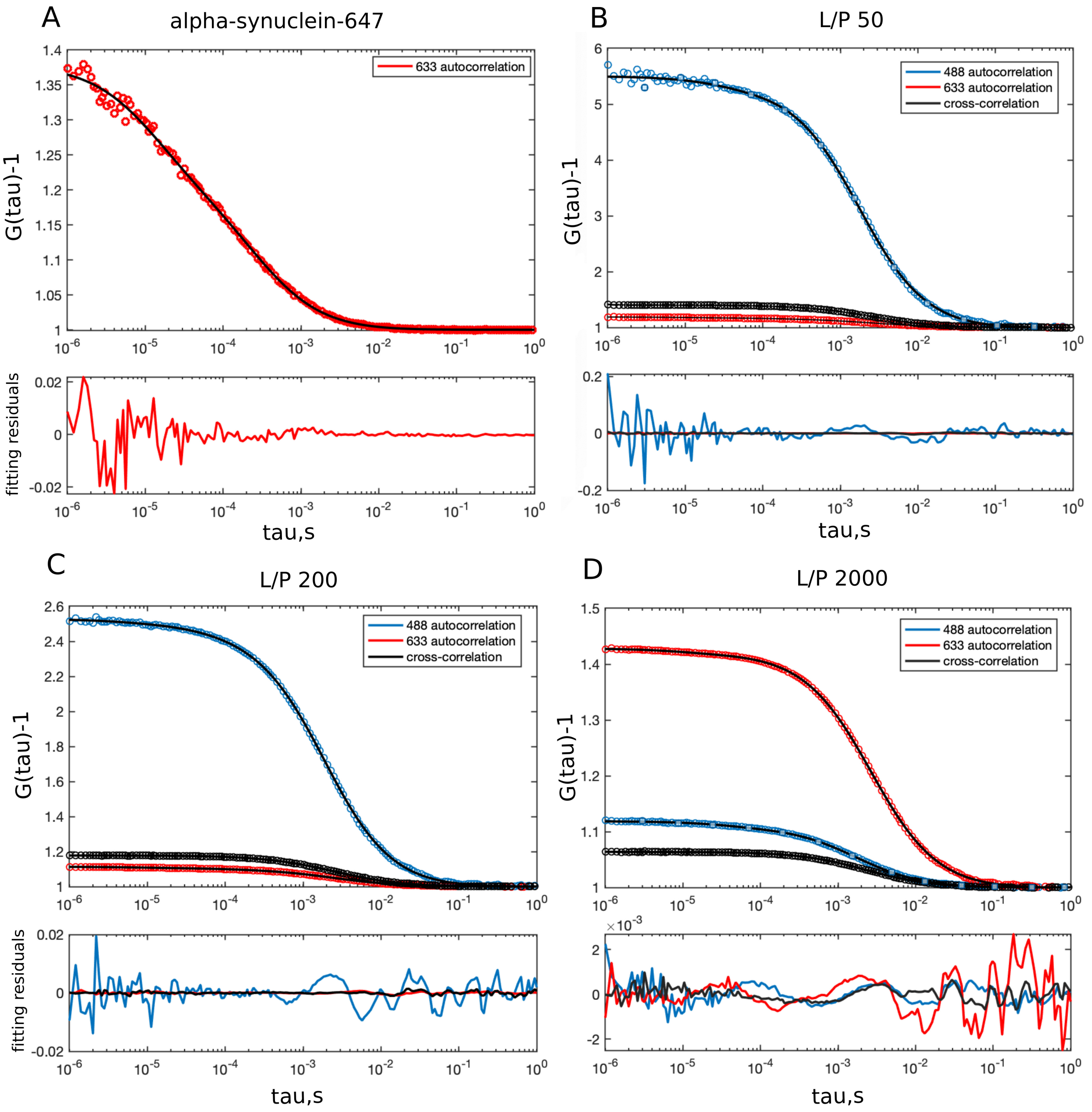

Figure S5: Fluorescence cross-correlation spectroscopy experimental results: A) Autocorrelation curve of free α-synuclein-647 B,C,D) 488 autocorrelation, 633 autocorrelation and cross-correlation curves for α-synuclein-647 with small unilamellar vesicles labelled with 0.5% Oregon Green DHPE at L/P 50, 200 and 2000, respectively. The experimental data (open circles) is plotted together with fits to a two-component model and fitting residuals.

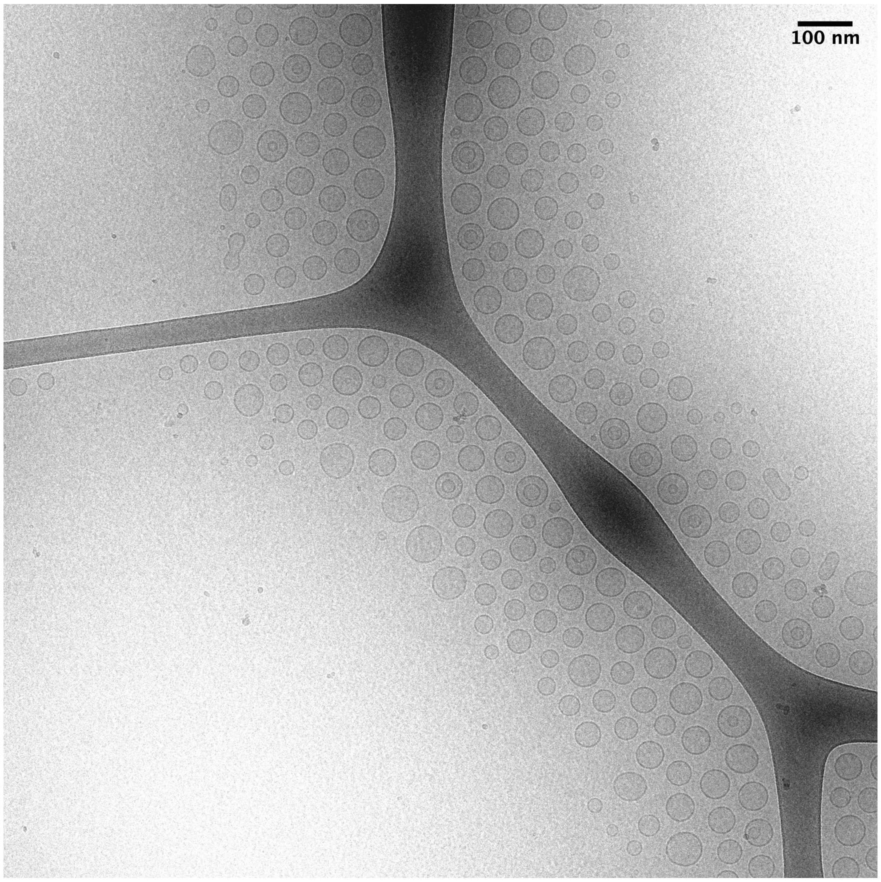

Figure S6: Cryo-TEM images of DOPC:DOPS 7:3 (mol) SUVs of 70 nm in diameter with no α-synuclein added.

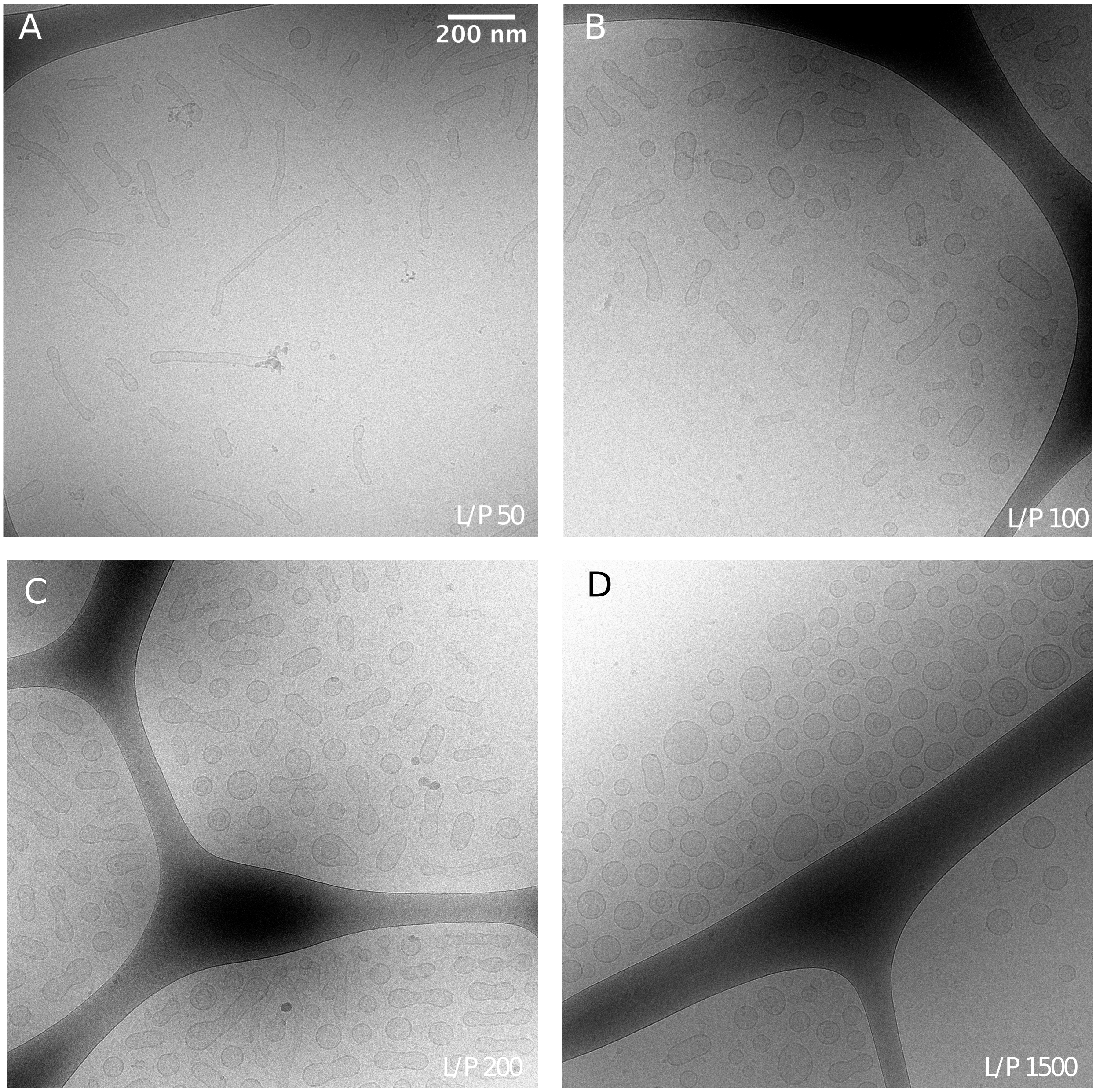

Figure S7: Cryo-TEM images of DOPC:DOPS 7:3 (mol) SUVs of 70 nm in diameter with α-synuclein at A) L/P=50, B) L/P=100, C) L/P=200 and D) L/P=1500.

Cryo TEM image analysis

For L/P 50, 100, 200, 400, 800, 1500 and 2000, the amount of deformed and non-deformed SUVs in the images was counted and the percentage of deformed vesicles was calculated as an average of results from 6 images. In this approach, the vesicles were assumed to deform

but not fuse, that is a long deformed vesicle was counted as one deformed vesicle. The results of this analysis are presented in Figure 4.

An alternative approach to the analysis was assuming fusion of SUVs and counting a fused vesicle with surface area corresponding to 3 vesicles as 3 vesicles (SI Figure S8). The percentage of deformed vesicles for each L/P estimated in this way are presented in SI Figure S9.

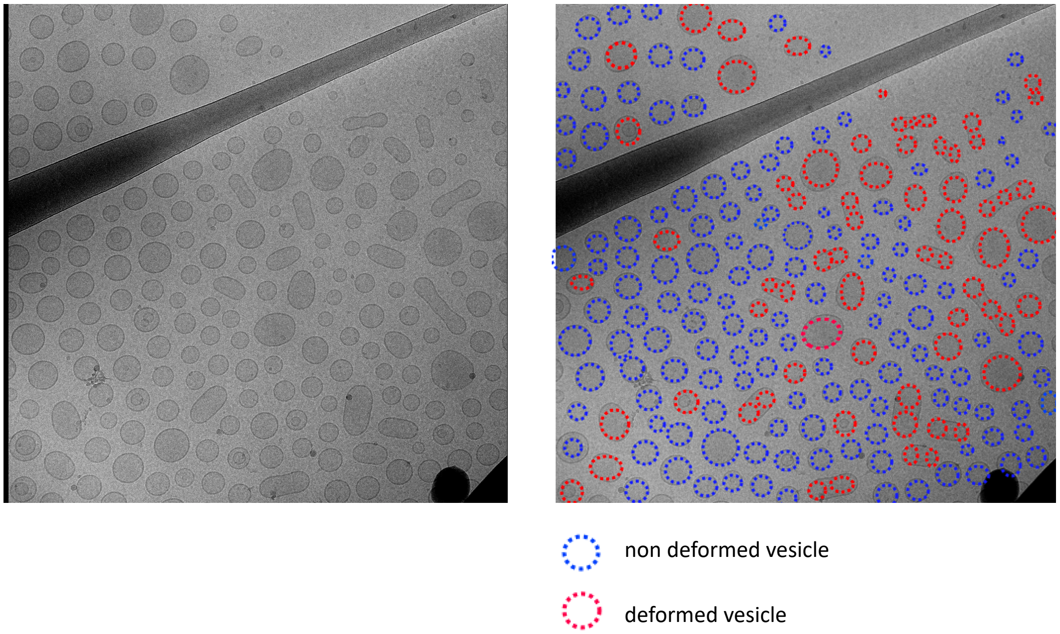

Figure S8: Cryo TEM image analysis: Sample image of α-synuclein with SUVs at L/P 2000. Non deformed and deformed vesicles were counted for 6 images for each sample. Fused vesicles clearly consisting from e.g. 3 vesicles were counted as 3 deformed vesicles. Fraction (%) of deformed vesicles was calculated for each L/P as an average of calculations from 6 images.

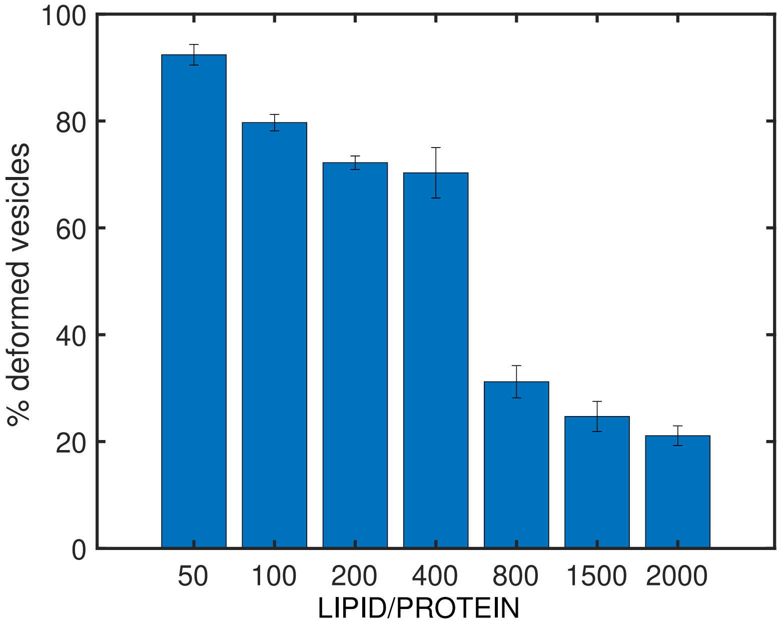

Figure S9: The percentage of deformed vesicles (mean ± SD) at different L/P ratios estimated from 6 different images of each sample from one experiment assuming that vesicles undergo α-synuclein induced fusion. The number of vesicles analyzed was 5330.
